# Neuroprotective Pathway Modulation by a Novel *Coriandrum sativum*, N-Acetylcysteine and Glutathione-Based Formulation: Insights from In Vitro 3D Models

**DOI:** 10.3390/ijms262210857

**Published:** 2025-11-08

**Authors:** Simone Mulè, Sara Ferrari, Rebecca Galla, Francesca Uberti

**Affiliations:** 1 Department for Sustainable Development and Ecological Transition, University of Piemonte Orientale (UPO), 13100 Vercelli, Italy; simone.mule@uniupo.it (S.M.); sara.ferrari@uniupo.it (S.F.); 2 Noivita Srls, Spin Off, University of Piemonte Orientale (UPO), Strada Privata Curti 7, 28100 Novara, Italy; rebecca.galla@uniupo.it

**Keywords:** natural supplements, nutraceuticals, antioxidant effect, in vitro 3D models, phytocomplex, neuroprotective potential

## Abstract

Pain remains a major clinical challenge due to its complex physiopathology and limited treatment options. In this context, several supplements based on palmitoylethanolamide (PEA) and alpha-lipoic acid (ALA) are known for their neuroprotective properties. ALA-based supplements have shown potential, but concerns about adverse effects persist. This study examines the formulations of two commercial products based on ALA and PEA, IperALA^®^ and IperALA^®^ Forte, in which ALA and vitamin D3 are replaced with *Coriandrum sativum* extract (*C. sativum* e.s.), N-acetylcysteine (NAC) and glutathione (GSH), assessing improvement of neuroprotective, anti-inflammatory and analgesic properties of the new formulation. Intestinal, blood–brain barrier (BBB), and central nervous system (CNS) models were sequentially stimulated with the test compounds. Both formulations were assessed for cytotoxicity, barrier integrity, permeability, oxidative stress, inflammation, and neuroprotection-related biomarkers. IperALA^®^ Forte demonstrated superior performance compared to IperALA^®^ and individual agents. It enhanced cell viability, preserved intestinal and BBB integrity, and improved compound permeability. Notably, it reduced ROS and pro-inflammatory cytokines (TNFα, IL-1), while increasing analgesic markers (CB2R, GABA) in the central system. The replacement of ALA and vitamin D3 with *C. sativum*, NAC, and GSH in IperALA^®^ Forte significantly improved the neuroprotective, antioxidant, and anti-inflammatory profile of the supplement. These results indicate a possible connection between the observed neuroprotective properties and the pathways involved in nociception and pain regulation, stating the hypothetical potential relevance of this approach for the treatment of pain-related conditions.

## 1. Introduction

Pain is a multifaceted experience with social, psychological, and biological components. Affecting roughly 33% of the general population, chronic pain frequently lasts past the typical healing period and is linked to comorbidities like anxiety, sleep disorders, and a lower quality of life, whereas nociceptive pain acts as a protective signal by indicating possible tissue damage [1,2,3].

Nociplastic pain has drawn more attention than other forms of chronic pain. It is believed to be caused by abnormal central nervous system (CNS) processing of pain signals and manifests without obvious tissue damage or peripheral nerve damage [4]. It is important to note that the severity of pain requires complicated activation of numerous brain areas responsible for sensory, affective, and cognitive processing, rather than being merely tied to the strength of peripheral inputs [5]. According to mechanistic research, central sensitisation, microglial activation, and neuroinflammation are important factors in the amplification and duration of pain [6,7]. Even though there are many different pharmacological and interventional treatments available, only a small percentage of patients receive adequate relief from chronic pain, and therapeutic choices frequently have limited outcomes [8,9]. Furthermore, prolonged usage of analgesics may result in negative side effects. As a result, there is now more interest in integrative methods that combine traditional therapies with nonpharmacological interventions, such as nutraceuticals [10,11,12,13].

Although the mechanisms behind many nutraceuticals remain partly unclear, growing evidence suggests their potential role in pain management and disease prevention [14]. By altering important receptors and pathways involved in both peripheral and central pain processing, such as opioid receptors, cannabinoid receptors (CB1R and CB2R), and Gamma-Aminobutyric Acid (GABA) receptors, nutraceuticals may help reduce pain through neuroprotective effects [15,16,17,18]. Additionally, by affecting mediators like prostaglandins, interleukin-1β (IL-1β), and tumor necrosis factor α (TNFα), and cellular components like microglia, which are becoming acknowledged as key participants in the regulation of pain within the CNS, they may have anti-inflammatory and neuroprotective effects [19,20]. These overlapping processes point to a possible dual function for nutraceuticals in pain management and neuroprotection.

Palmitoylethanolamide (PEA) is fundamental in this context, as it has a structure similar to that of endocannabinoids. It is a substance that affects the mechanisms of neuropathic pain and analgesics, acting on immune cells, neurons, and microglia [21]. It therefore acts in a complex manner, exhibiting a wide range of pharmacological actions that target different receptors, such as CB1R and CB2R, as well as inflammatory cytokines [22,23,24]. Some studies prove this; for example, in one study, it was observed that the association of PEA with tramadol had a synergistic effect. The combination resulted in a better response or a relative reduction in tramadol dosage [25,26]. Similar effects have been found in the extracts of many plant essential oils, including those from *Cannabis sativa*, *Syzygium aromaticum*, *Piper nigrum*, *Rosmarinus officinalis*, and *Origanum vulgare* L., which include the bicyclic sesquiterpene β-caryophyllene (BCP) [18,27]. BCP has demonstrated antinociceptive effects in experimental models, both alone and in combination with opioids, and has been shown to reduce inflammatory cytokines in diabetic neuropathy models [28,29,30].

In addition, *Coriandrum sativum* L. (*C. sativum*), which belongs to the family *Apiaceae,* has also become relevant for the treatment of neuropathic pain. Due to its antioxidant capacity, *C. sativum* is recognised as one of the strongest food antioxidants. Among its intriguing pharmacological properties are anti-inflammatory, anti-dyslipidaemia, cardiovascular and neuroprotective effects [31]. Oral administration of the bioactive components of *C. sativum* significantly alleviated oxidative–nitrosative stress, reduced pain threshold and decreased hyperglycaemia in diabetic rats. The flavonoids contained in *C. sativum* show significant affinity for the TNFα binding site, according to a linkage study [32].

Based on the scientific evidence previously described, our research focused on examining two commercial product formulations (IperALA^®^ and IperALA^®^ Forte, Pharma Suisse Laboratories SpA, Milan, Italy) that contain alpha-lipoic acid (ALA), suitable for supporting nerve function, and PEA and BCP, which can modulate analgesic responses. ALA’s ability to act in both aqueous and lipid environments makes it particularly effective in countering oxidative damage and neuroinflammation associated with conditions such as neuropathic pain. To date, however, ALA has been the subject of investigation by the scientific community due to some possible adverse effects reported in the context of glycaemia and at the gastrointestinal and cutaneous levels [33,34,35].

Therefore, based on the above, this study focused on the biological effects of the components of the formulations (IperALA^®^ and IperALA^®^ Forte) for oral use, which were also selected based on the pharmacokinetic properties of the ingredients. Both contain micronised palmitoylethanolamide (PEA-m), which improves oral absorption and selectively accumulates in inflamed tissues, including the CNS, where it modulates inflammation and pain through peroxisome proliferator-activated receptor α (PPAR-α) and the endocannabinoid system [36,37,38]. BCP is also present, known for its rapid absorption and ability to cross the blood–brain barrier (BBB), acting on neuroinflammatory pathways [39]. IperALA^®^ also includes ALA and vitamin D3, both of which are well-absorbed and able to penetrate the CNS [40,41,42,43]. In contrast, IperALA^®^ Forte replaces the latter with N-acetylcysteine (NAC) and glutathione (GSH), which support antioxidant defences and can cross the BBB to varying degrees, and in combination, they show better neuroprotective, anti-inflammatory and antioxidant capacities [44,45,46]. In addition, IperALA^®^ Forte also contains CORILOL^®^, a standardised extract of *C. sativum* rich in antioxidant phytochemicals with neuroprotective effects and good permeability to the BBB [47,48]. This composition supports their potential in combatting oxidative stress (OS), neuroinflammation, and neuroprotection pathway modulation in the CNS.

Taken together, these features support a mechanistically justified in vitro model simulating the physiological absorption route from the intestinal barrier to the brain compartment, allowing for a realistic assessment of the formulations’ multi-level biological effects. Indeed, the present study aimed to investigate, through advanced 3D in vitro models mimicking the intestinal barrier, the BBB, and the CNS, the biological effects of IperALA^®^ and IperALA^®^ Forte as well as their individual bioactive components. Similar to another study [49], the neuroprotective, antioxidant, anti-inflammatory, and preliminary analgesic-related mechanisms of IperALA^®^ Forte were evaluated in comparison with the current ALA and vitamin D3-based formulation (IperALA^®^). The objective was to assess whether the replacement of ALA and vitamin D3 could enhance the mechanistic profile of the supplement, with a focus on biomarkers involved in OS, inflammation, and central nervous system neuroprotection modulation with preliminary data in the context of nociception (CB2R and GABA).

## 2. Results

### 2.1. Evaluation of the Biological Effects of Formulations and Their Components at the Level of an In Vitro 3D Intestinal Barrier Model

**Figure 1:**
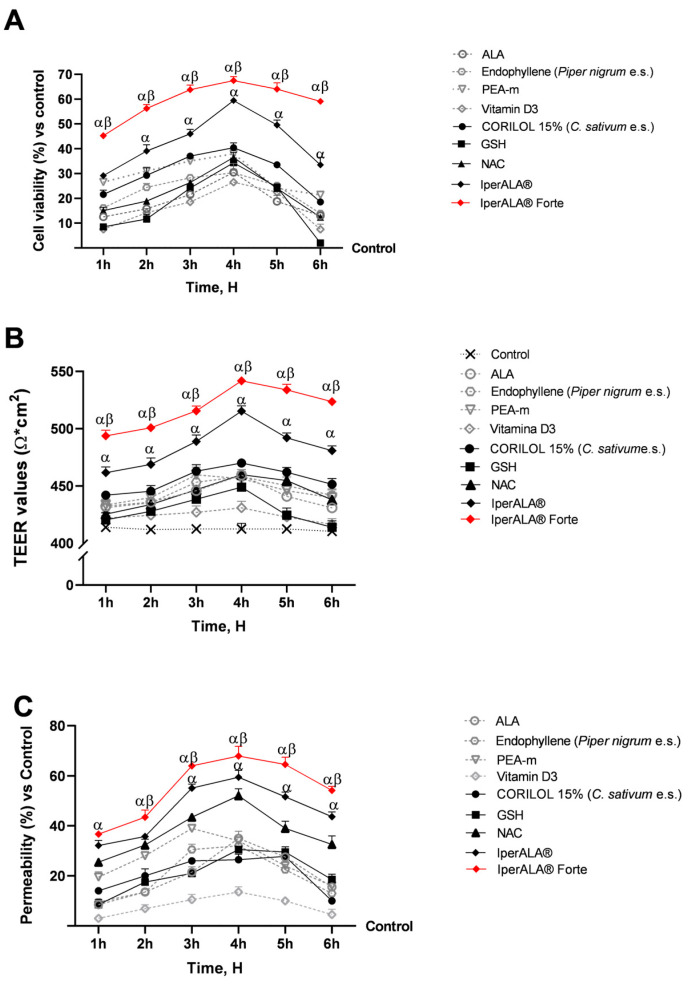
Results after 1–6 h of treatment of the samples under examination at the level of an in vitro 3D model of the intestinal barrier. (**A**) Cell viability obtained by MTT assay; (**B**) TEER values measured by the EVOM3™ instrument; (**C**) permeability rate examined by fluorescent probe. Data are expressed as mean ± SD (%) of 5 independent experiments performed in triplicate, normalised to the control (0% line only in (**A**,**C**)) to highlight treatment-related changes. Positive values indicate increases relative to the control. α *p* < 0.05 vs. single agents; β *p* < 0.05 vs. IperALA^®^.

Experiments were conducted using a 3D Transwell^®^ model of the intestinal compartment with Caco-2 cells to assess the potential cytotoxic effects of the test samples. This preliminary evaluation was essential to verify cell viability and the preservation of epithelial integrity. As can be seen in Figure 1A,B, the mature intestinal epithelium was stimulated for 1 h–6 h by treatment at the apical level with the complete formulations (IperALA^®^ and IperALA^®^ Forte) and the individual components, dissolved in 1 mL of DMEM culture medium as indicated in Section 4.1 and diluted 2000 times with respect to the human dosage reported in Table 1. All samples were shown to maintain a state of cell viability with a peak around 4 h of treatment. In detail, as shown in Figure 1A, both IperALA^®^ and IperALA^®^ Forte were found to increase the cell viability of the individual components in a statistically significant manner (*p* < 0.05). In addition, the formulation of IperALA^®^ Forte, following the replacement of ALA and vitamin D3 with CORILOL 15% (*C. sativum* e.s.), NAC, and GSH, yielded significantly improved outcomes compared to IperALA^®^. Specifically, a 15% higher peak in cell viability was recorded at 4 h of treatment. This enhancement reflects a quantitatively greater effect size, and the difference was confirmed to be statistically significant (*p* < 0.05) based on appropriate inferential analyses (ANOVA or Student’s *t*-test) and analysis of the η^2^ value (η^2^ = 0.20), indicating that the observed improvement is statistically robust and not attributable to random variability. In addition, IperALA^®^ Forte improved the percentage effect by 55% compared to ALA, 40% compared to CORILOL 15% (*C. sativum* e.s.), 46% compared to NAC, 49% compared to GSH, 55% compared to endophyllene (*Piper nigrum* e.s.), 78% compared to PEA-m, 61% compared to vitamin D3, and 15% compared to IperALA^®^. Among the new ingredients added as replacements for ALA and vitamin D3, CORILOL 15% (*C. sativum* e.s.) performed the best in comparison with the others.

During this phase, precise measurements of the intestinal epithelium-associated TEER value (Figure 1B) were taken across the treatment period (1–6 h) using EVOM3™. This parameter was essential to assess the integrity and functional maintenance of the intestinal epithelium. As shown in Figure 1B, the measured TEER values associated with all tested samples were higher than those of the control (>400 Ω∙cm^2^; *p* < 0.05), positively influencing the state of intestinal barrier integrity. Consistent with the data on cell viability (Figure 1A), IperALA^®^ Forte yielded the best results, with a peak TEER at 4 h of 544 Ω × cm^2^ (*p* = 0.0002 with large effect size η^2^ = 0.33 compared to IperALA^®^). Among the individual agents, CORILOL 15% (*C. sativum* e.s.) gave the highest TEER results compared to vitamin D3 (*p* < 0.05), similar to ALA. Compared to vitamin D3, both NAC and GSH individually exhibited significantly higher TEER values (*p* < 0.05), specifically between 3 and 4 h for GSH and between 3 and 6 h for NAC.

Following the absence of cytotoxic effects and epithelial damage, the permeability of specific agents and formulations was further assessed over a 1–6 h treatment period using a fluorescent probe. As shown in Figure 1C, IperALA^®^ and IperALA^®^ Forte were found to enhance the absorption kinetics of individual agents while increasing the permeability of IperALA^®^ (*p* < 0.05 with medium effect size η^2^ = 0.06). Except for PEA-m and endophyllene (*Piper nigrum* e.s.), all samples exhibited peak absorption at approximately 4 h of treatment. Among the individual agents, NAC yielded the best results in terms of permeability, with a value of 51% compared to the control at 4 h (*p* < 0.05). GSH and CORILOL 15% (*C*. *sativum* e.s.) showed greater absorption compared to vitamin D3 alone throughout the entire observation period. Statistically significant differences (*p* < 0.05) were observed starting at 2 h of treatment and persisted up to 5 h for CORIOL 15% and 6 h for GSH. In comparison, ALA exhibited permeability values comparable to those of GSH and CORILOL 15% (*C. sativum* e.s.), with no significant differences among them. IperALA^®^ Forte improved the permeability percentage increase rate around peak absorption through the intestinal epithelium by 49% compared to ALA, 59% compared to CORILOL 15% (*C. sativum* e.s.), 26% compared to NAC, 56% compared to GSH, 54% compared to endophyllene (*Piper nigrum* e.s.), 43% compared to PEA-m, 79% compared to vitamin D3, and 15% compared to IperALA^®^.

### 2.2. Assessment of the Effects of Both Single and Combined Substances on a 3D In Vitro BBB Model Following Intestinal Metabolisation

**Figure 2:**
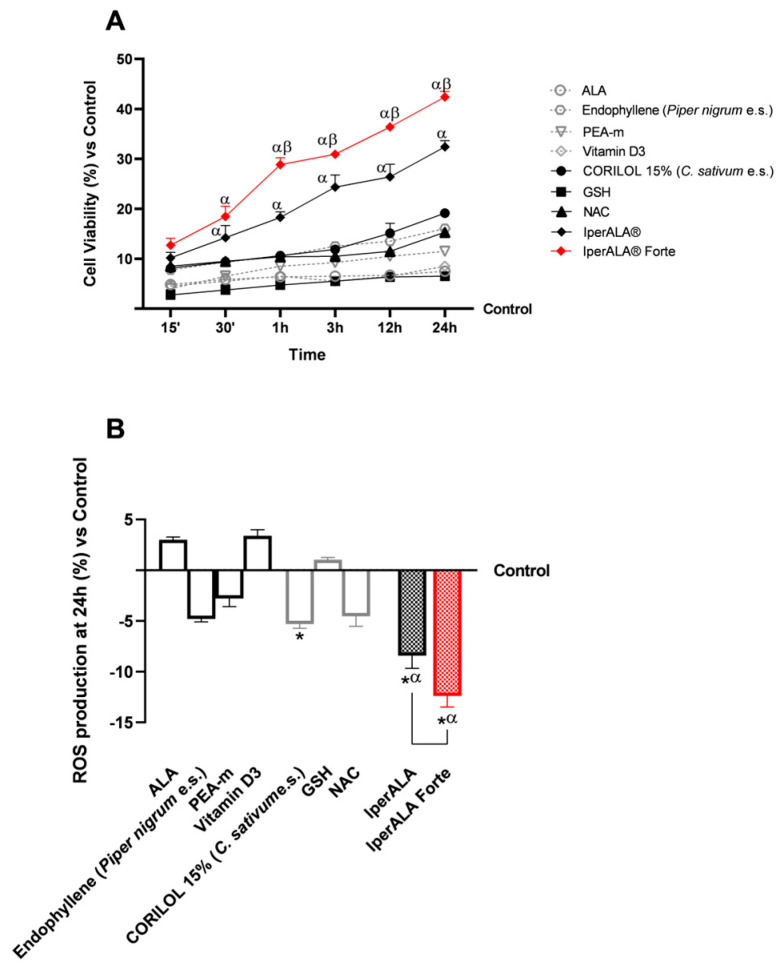
Results obtained after treatment of the test samples at the level of a 3D in vitro model of BBB. In (**A**), cell viability was obtained by an MTT assay at 15′–30′–1 h–3 h–12 h–24 h; in (**B**), ROS production was measured by an assay with cytochrome C after 24 h of treatment. Data are expressed as mean ± SD (%) of 5 independent experiments performed in triplicate, normalised to the control (0% line) to highlight treatment-related changes. Positive and negative values indicate increases or decreases, respectively, relative to the control. For (**A**): α *p* < 0.05 vs. single agents; β *p* < 0.05 vs. IperALA^®^. For (**B**): * *p* < 0.05 vs. control; α *p* < 0.05 vs. single agents; the bar indicates *p* < 0.05 vs. IperALA^®^.

Following stimulation of the 3D intestinal barrier model, the basolateral supernatant, containing metabolites that passed through the intestinal epithelium, was collected from each test sample. To conduct fundamental analyses of the state of maintenance and cell viability increase (Figure 2A) with individual components and combinations at the level of the BBB model, the production of reactive oxygen species (ROS), key indicators for examination, was investigated. As shown in Figure 2A, following metabolisation at the intestinal level, all tested samples exhibited a time-dependent percentage rate increase in cell viability, peaking around 24 h of treatment, with significant values compared to the control (*p* < 0.05). Among the individual new ingredients added as ALA and vitamin D3 substitutes, CORILOL 15% (*C. sativum* e.s.) and NAC performed better, with viability rates greater than 8%, than ALA and vitamin D3 (<7%). GSH, however, exhibited absorption values comparable to those of ALA and vitamin D3 formulations, although slightly lower in the 15’ to 3 h interval. IperALA^®^ and IperALA^®^ Forte showed a marked percentage increase in cell viability with statistically significant values compared to the individual agents (*p* < 0.05). IperALA^®^ Forte, without ALA and vitamin D3, yielded the best results by enhancing the effectiveness of the original formulation. Specifically, IperALA^®^ Forte increased the percentage peak viability by 82% over ALA, 55% over CORILOL 15% (*C. sativum* e.s.), 64% over NAC, 85% over GSH, 62% over endophyllene (*Piper nigrum* e.s.), 73% over PEA-m, 80% over vitamin D3, and 26% over IperALA^®^ (*p* < 0.04 with medium effect size η^2^ = 0.12).

Similar data were also obtained in terms of OS (Figure 2B). All samples evaluated allowed ROS levels to be maintained within physiological limits, consistent with the data on cell viability compared to the control. As shown in Figure 2B, among the individual agents, the best antioxidant effects were expressed by CORILOL 15% (*C. sativum* e.s.; *p* < 0.05), NAC (*p* < 0.05) and endophyllene (*Piper nigrum* e.s.), not always statistically significant compared to the control but with significant effects in comparison with ALA, vitamin D3, and GSH (*p* < 0.05). Both formulations examined allowed for improvement of the effects of individual agents (*p* < 0.05), with a better antioxidant potential by IperALA^®^ Forte without ALA and vitamin D3 compared to IperALA^®^ (*p* = 0.0004 with large effect size η^2^ = 0.76). IperALA^®^ Forte increased the antioxidant percentage effect by 3 times compared to ALA, 56% compared to CORILOL 15% (*C. sativum* e.s.), 63% compared to NAC, 91% compared to GSH, 60% compared to endophyllene (*Piper nigrum* e.s.), 77% compared to PEA-m, 2.5 times compared to vitamin D3, and 30% compared to IperALA^®^.

**Figure 3:**
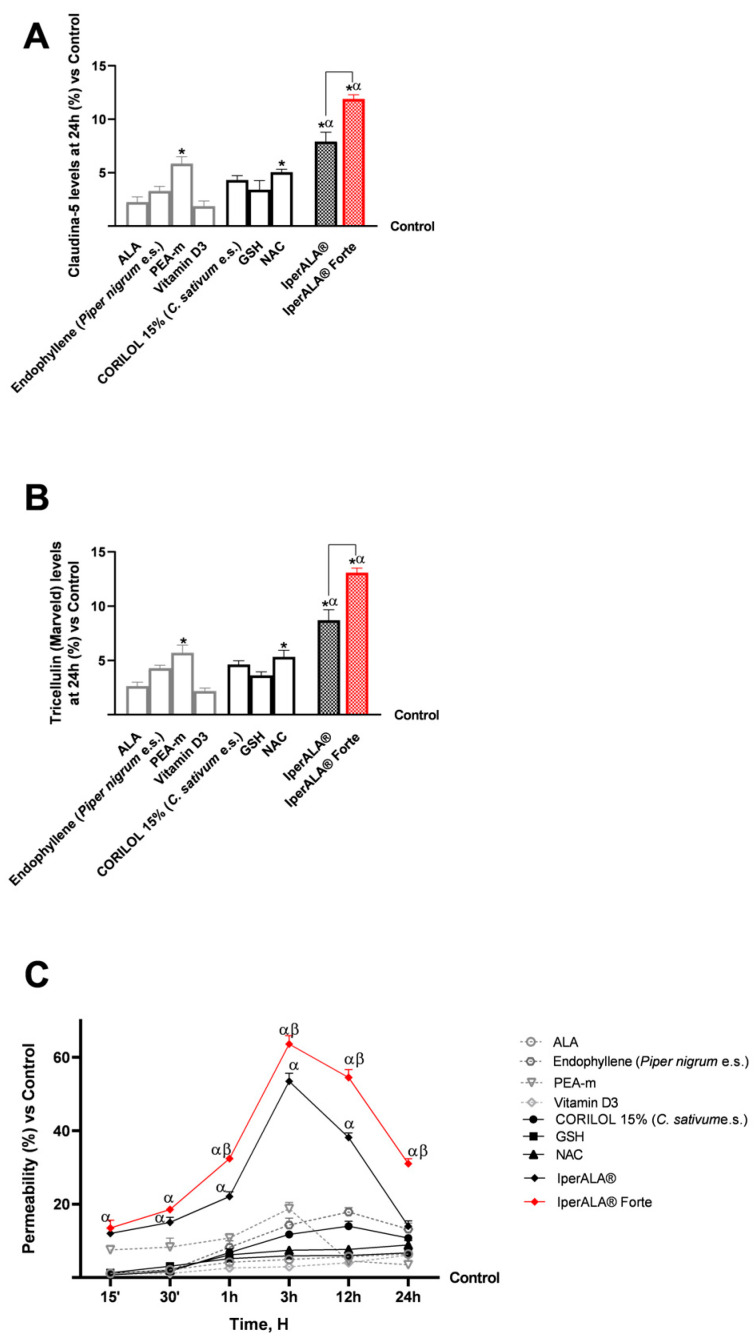
Results obtained after treatment with the test samples at the level of a 3D in vitro model of the BBB. (**A**) Claudin-5 levels obtained by an ELISA kit at 24 h; (**B**) marveld (or tricelluline) levels obtained by an ELISA kit at 24 h; (**C**) permeability rate examined by a fluorescent probe. Data are expressed as mean ± SD (%) of 5 independent experiments performed in triplicate, normalised to the control (0% line) to highlight treatment-related changes. Positive values indicate increases relative to the control. For (**A**,**B**): * *p* < 0.05 vs. control; α *p* < 0.05 vs. single agents; the bar indicates *p* < 0.05 vs. IperALA^®^. For (**C**): α *p* < 0.05 vs. single agents; β *p* < 0.05 vs. IperALA^®^.

**Figure A1:**
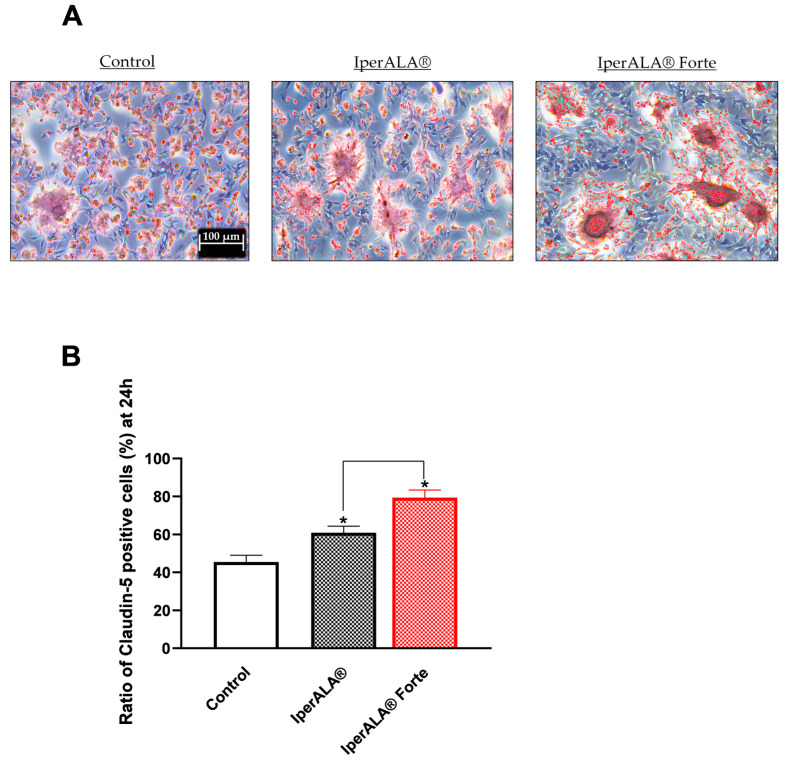
Analysis of claudin-5 in terms of positive cells by immunocytochemistry on BBB tri-culture model after 24 h treatment with IperALA^®^ and IperALA^®^. (**A**) Representative images for each sample obtained with microscope at 10× (100 μm) objective after immunocytochemistry protocol; (**B**) % score of positive cells counted in 12 different areas following treatment, obtained with ImagePro 3 software. * *p* < 0.05 vs. control; the bar indicates *p* < 0.05 vs. IperALA^®^.

The study then focused on the effects of individual agents and formulations on barrier integrity and functionality (Figure 3), through the analysis of key tight junction (TJ) proteins such as claudin-5 and tricellulin (marveld). As shown in Figure 3A,B, after 24 h of treatment, all samples exhibited no alterations in TJ levels, with values higher than those of the control (*p* < 0.05). As shown in Figure 3A,B, looking at the individual agents, the most significant results compared to the control were obtained after stimulation with PEA-m and NAC (*p* < 0.05) and in a similar way also by CORILOL 15% (*C. sativum* e.s.). IperALA^®^ Forte, with the substitution of ALA and vitamin D3, resulted in a statistically significant improvement in the biological action of IperALA^®^ (for claudin-5, *p* = 0.0005 with large effect size η^2^ = 0.84; for marveld, *p* = 0.0001 with large effect size η^2^ = 0.89). Both formulations gave the most significant results compared to the individual agents, increasing their percentage effect at the level of the BBB, according to the data obtained in terms of viability and OS (*p* < 0.05). Through immunohistochemistry, additional data were obtained on the expression of claudin-5 in the same experimental conditions following treatment with IperALA^®^ and IperALA^®^ Forte. The results in Appendix A (Figure A1) are also represented by images obtained under the microscope and by the determination of the density of positive cells using ImagePro 3 software. Immunocytochemistry data outline a greater percentage effect on claudin-5 expression following both treatments (IperALA^®^ and IperALA^®^ Forte) with most significant data from IperALA^®^ Forte compared to IperALA^®^ (*p* < 0.05).

Finally, as performed at the intestinal level, the analyses focused on the evaluation of the permeability rate of the samples, examined using a fluorescent probe along the BBB model (Figure 3C). All the various compounds exhibited the same absorption profile over the time studied, with a maximum permeability at approximately 12 h of stimulation, except for PEA-m, which peaked at approximately 3 h. PEA-m, endophyllene (*Piper nigrum* e.s.), and CORILOL 15% (*C. sativum* e.s.) showed the highest permeability across the in vitro BBB model compared to the other single agents, especially ALA and vitamin D3, from 1 to 24 h of analysis and from 15′ as regards PEA-m. The permeability rate of individual components across the BBB was statistically significantly improved by both formulations, IperALA^®^ and IperALA^®^ Forte, during the treatment period (*p* < 0.05), with a maximum absorption at 3 h of stimulation (63.6% by IperALA^®^ Forte vs. 53.5% by IperALA^®^ with *p* < 0.05 with medium effect size η^2^ = 0.07). More relevantly, starting from 1 h of stimulation, more significant results were outlined by IperALA^®^ Forte compared to IperALA^®^, with a greater statistical significance gap (*p* < 0.05). In detail, IperALA^®^ Forte enhanced the permeability percentage rate around the absorption peak by 92% compared to ALA, 80% compared to CORILOL 15% (*C. sativum* e.s.), 87% compared to NAC, 90% compared to GSH, 75% compared to endophyllene (*Piper nigrum* e.s.), 71% compared to PEA-m, 95% compared to vitamin D3, and 16% compared to IperALA^®^.

### 2.3. Analysis of Biological Effects of Single Substances and Formulations at the Level of a Brain Organoid Model After Crossing the BBB

The third phase of the study focused on the CNS, using a cerebral organoid model pre-treated with 200 μM H_2_O_2_ for 30 min to induce neuroinflammation [50]. The organoids were then treated for 24 h with the basolateral supernatant collected from the Transwell^®^ BBB model exposed to each test sample.

**Figure 4:**
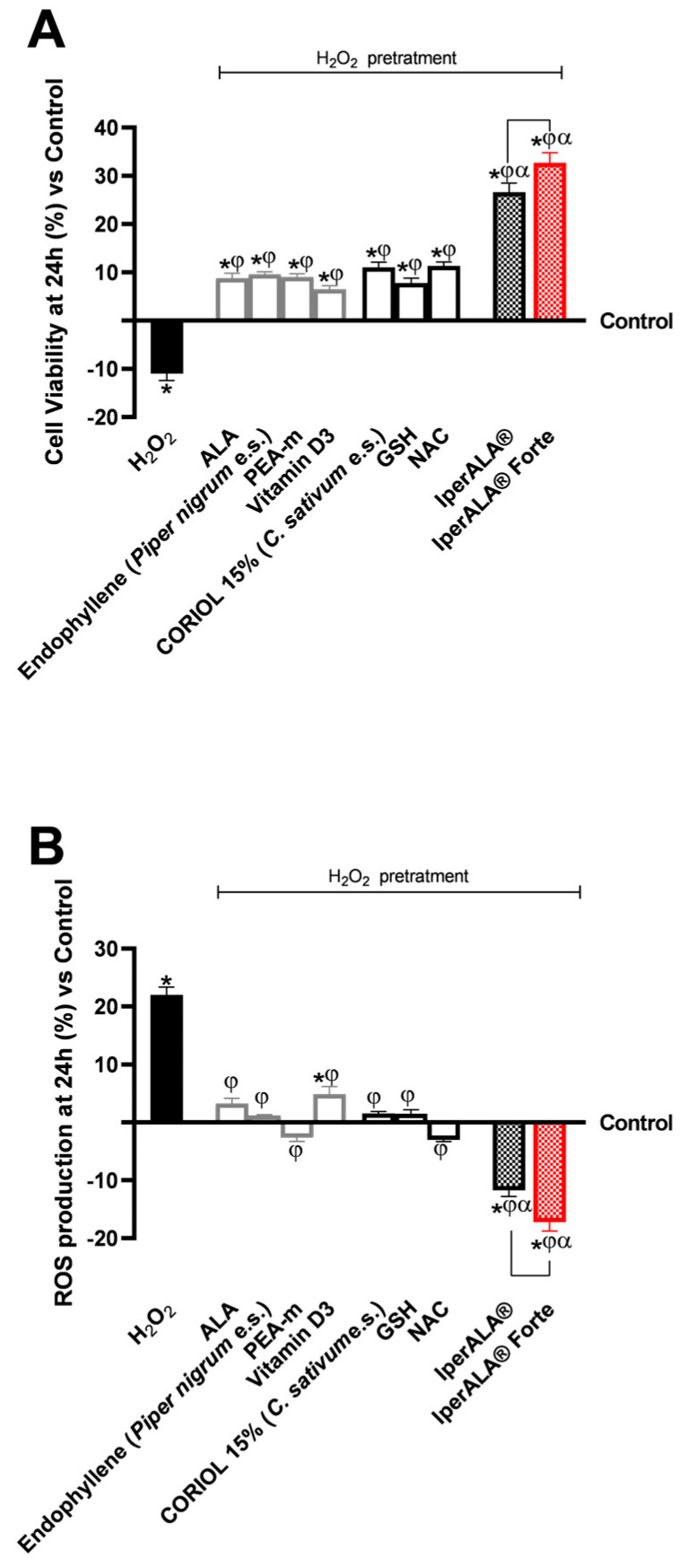
Results obtained after treatment for 24 h with test samples at the level of an in vitro model of cerebral organoids under conditions of neuroinflammation and OS after pre-treatment with H_2_O_2_ 200 μM. In (**A**), cell viability was assessed by the MTT assay at 24 h; in (**B**), ROS production was measured using the cytochrome C assay after 24 h of treatment. Data are expressed as mean ± SD (%) of 5 independent experiments performed in triplicate, normalised to the control (0% line) to highlight treatment-related changes. Positive and negative values indicate increases or decreases, respectively, relative to the control. * *p* < 0.05 vs. control; φ *p* < 0.05 vs. H_2_O_2_ 200 μM; α *p* < 0.05 vs. single agents; the bar indicates *p* < 0.05 vs. IperALA^®^.

For cytotoxicity analyses on cerebral organoids, viability analysis was performed by the MTT assay (Figure 4A) and OS analysis by the cytochrome C method (Figure 4B). In all the analyses performed, pre-treatment with H_2_O_2_ 200 μM promoted a reduction in cell viability (11% less than the control; *p* < 0.05) and, simultaneously, an increase in ROS production above physiological levels (22% more than the control; *p* < 0.05). As shown in Figure 4A, both formulations enhanced the protective effect on cell viability compared to the individual agents (*p* < 0.05). IperALA^®^ Forte also demonstrated a significantly greater percentage increase in viability: 19% higher than IperALA^®^ (*p* = 0.005 with large effect size η^2^ = 0.71). In addition, compared to H_2_O_2_ 200 μM, IperALA^®^ Forte improved the percentage cell viability by 1.3 times (*p* < 0.05). Analysing the effects of individual agents, the cell viability rate was increased compared to the control, counteracting the neurotoxic effect of H_2_O_2_ 200 μM; the best effects were obtained by CORILOL 15% (*C. sativum* e.s.) and NAC, with statistically significant results compared to vitamin D3.

Characterisation of the levels of ROS produced during the analyses in this phase allowed us to confirm the results obtained on the cell viability of the cerebral organoid. IperALA^®^ and IperALA^®^ Forte formulations (Figure 4B) helped to eliminate the pro-oxidant effect promoted by H_2_O_2_ 200 μM by amplifying the percentage antioxidant effect of individual components (*p* < 0.05). The replacement of ALA and vitamin D3 in IperALA^®^ Forte with new bioactive components increased the antioxidant effect at the neuronal level by 35% compared IperALA^®^ (*p* = 0.002 with large effect size η^2^ = 0.77), reducing the strongly oxidative action of H_2_O_2_ 200 μM by about 1.6 times compared to IperALA^®^. In this context, among the new ingredients added to IperALA^®^ Forte, NAC gave statistically significant results compared to all other single agents, similar to PEA-m (*p* < 0.05).

In addition to the antioxidant potential, further analysis was carried out on the anti-inflammatory effects of the formulations under consideration and their modulation in the neuroprotection context.

**Figure 5:**
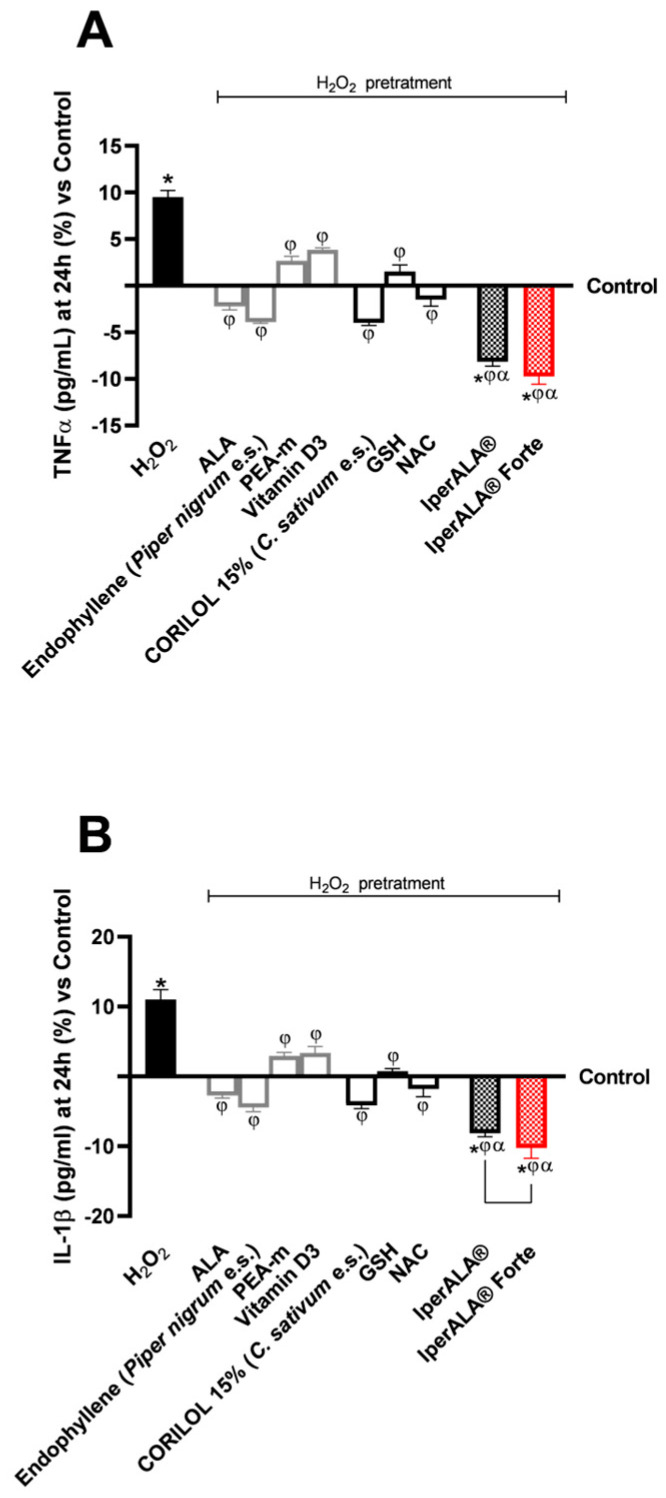
Results obtained on the inflammatory process after treatment for 24 h with test samples at the level of an in vitro model of a brain organoid under conditions of neuroinflammation and OS after pre-treatment with H_2_O_2_ 200 μM. (**A**) TNFα production (pg/mL) obtained by ELISA kit at 24 h; (**B**) IL-1β production (pg/mL) obtained by ELISA kit at 24 h. Data are expressed as mean ± SD (%) of 5 independent experiments performed in triplicate, normalised to the control (0% line) to highlight treatment-related changes. Positive and negative values indicate increases or decreases, respectively, relative to the control. * *p* < 0.05 vs. control; φ *p* < 0.05 vs. H_2_O_2_ 200 μM; α *p* < 0.05 vs. single agents; the bar indicates *p* < 0.05 vs. IperALA^®^.

Regarding inflammation, this study focused on the rate of production of two crucial pro-inflammatory cytokines, TNFα (Figure 5A) and IL-1β (Figure 5B), following treatment with the test samples. For both cytokines examined, pre-treatment with 200 μM H_2_O_2_ promoted a percentage increase in levels compared to the control condition, with 10% for TNFα and 12% for IL-1β, respectively. All individual agents examined showed an active role in modulating the context of H_2_O_2_ 200 μM-induced neuroinflammation (*p* < 0.05), with better effects from endophyllene (*Piper nigrum* e.s.) and CORILOL 15% (*C. sativum* e.s.). Both IperALA^®^ and IperALA^®^ Forte formulations’ effects were amplified by the combined action of individual agents, which improved the percentage inflammatory profile (*p* < 0.05), with more significant data from IperALA^®^ Forte than the old IperALA^®^ formula (*p* < 0.05, except for the production of TNFα in Figure 5A). The effect size for neuroinflammation of IperALA^®^ Forte compared to IperALA^®^ was larger (η^2^ = 0.43 for TNFα and η^2^ = 0.36 for IL-1β) but only statistically significant for IL-1β levels (*p* < 0.05). Analysing the individual agents, all showed an active role in modulating H_2_O_2_ 200 μM-induced neuroinflammation (*p* < 0.05), with better effects from endophyllene (*Piper nigrum* e.s.) and CORILOL 15% (*C. sativum* e.s.). At the same time, optimal rates of neurotoxic action of H_2_O_2_ 200 μM were obtained from ALA and NAC, but they were lower than endophyllene (*Piper nigrum* e.s.) and CORILOL 15% (*C. sativum* e.s). IperALA Forte improved the percentage decrease rate of production of TNFα in pg/mL by 2 times compared to H_2_O_2_ 200 μM, 76% compared to ALA, 59% compared to CORILOL 15% (*C. sativum* e.s.), 85% compared to NAC, 1.2 times compared to GSH, 60% compared to endophyllene (*Piper nigrum* e.s.), 71% compared to PEA-m, 1.2 times compared to vitamin D3 and 16% compared to IperALA^®^. For the production of IL-1β in pg/mL, IperALA^®^ Forte improved the percentage decrease rate by 2.1 times compared to H_2_O_2_ 200 μM, 73% compared to ALA, 60% compared to CORILOL 15% (*C. sativum* e.s.), 82% compared to NAC, 1.1 times compared to GSH, 57% compared to endophyllene (*Piper nigrum* e.s.), 1.3 times compared to PEA-m, 1.3 times compared to vitamin D3 and 20% compared to IperALA^®^.

The analysis was extended to key molecular targets associated with neuroinflammation, including CB2R and GABA receptors, in the brain organoid model. In all parameters examined, pre-treatment with H_2_O_2_ 200 μM promoted the onset of a neurotoxic and hypothetically pro-nociceptive context by inhibiting CB2R levels, and at the same time significantly inhibited the presence of GABA compared to the control condition (*p* < 0.05). All samples examined were able to counteract the H_2_O_2_ 200 μM-induced algesia condition (*p* < 0.05). Both formulations, IperALA^®^ and IperALA^®^ Forte, gave the best percentage of biological effects compared to single agents for all biological markers examined (*p* < 0.05). Substitution of ALA and vitamin D3 in IperALA^®^ Forte helped to improve the percentage of the hypothetical analgesic profile of the IperALA^®^ formulation (*p* < 0.05) by increasing the presence of CB2R and bringing GABA levels up to control levels with statistically significant data compared to IperALA^®^. The effect size of CB2R and GABA levels was large, with η^2^ = 0.73 (*p* = 0.004) and η^2^ = 0.88 (*p* < 0.0001), respectively. Indeed, IperALA^®^ Forte increased the percentage levels of CB2R and GABA by 33% and 1.75 times compared to IperALA^®^ and 13 times compared to H_2_O_2_ 200 μM, respectively.

**Figure 6:**
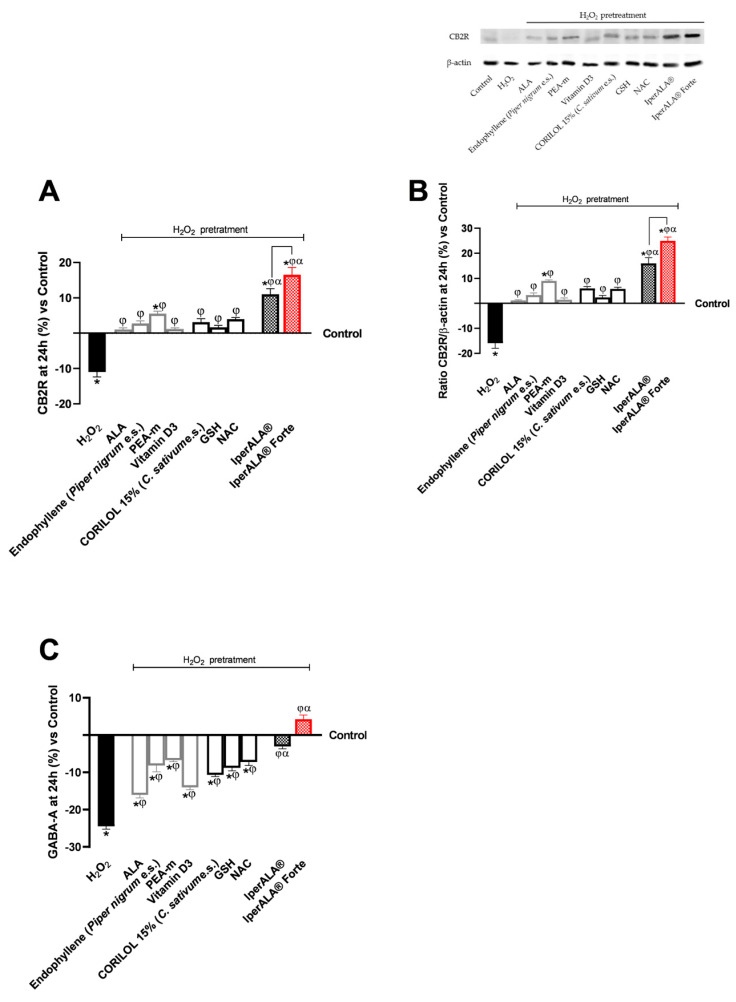
Results obtained on biological markers related to neuroinflammation pathways and the context of nociception, after treatment for 24 h with test samples at the level of an in vitro brain organoid model under conditions of pre-treatment with H_2_O_2_ 200 μM. (**A**) CB2R levels obtained by an ELISA kit at 24 h; (**B**) CB2R densitometric analysis after Western Blot, which is reported as an example image; (**C**) GABA levels obtained by ELISA kit at 24 h. Data are expressed as mean ± SD (%) of 5 independent experiments performed in triplicate, normalised to the control (0% line) to highlight treatment-related changes. Positive and negative values indicate increases or decreases, respectively, relative to the control. * *p* < 0.05 vs. control; φ *p* < 0.05 vs. H_2_O_2_ 200 μM; α *p* < 0.05 vs. single agents; the bar indicates *p* < 0.05 vs. IperALA^®^.

Examining the individual agents on CB2R, the most statistically significant effect, also compared to the control (*p* < 0.05), was promoted by PEA-m with similar but lower results obtained by CORILOL 15% (*C. sativum* e.s.) and NAC, comparable to endophyllene (*Piper nigrum* e.s.). The findings from the CB2R ELISA kits (Figure 6A) were verified by using the Western Blot technique and densitometric analysis. As shown in Figure 6B, all individual agents reported CB2R expression levels and densitometric analysis results close to those of the untreated control (0% line; *p* < 0.05 only for PEA-m) with statistically significant data compared to H_2_O_2_ 200 μM (*p* < 0.05). Both formulations, IperALA^®^ and IperALA^®^ Forte, gave the best biological percentage effects compared to individual agents in a statistically significant manner (*p* < 0.05). IperALA^®^ Forte increased the biological effect of CB2R expression by 33% compared to the IperALA^®^ formulation (*p* = 0.0002 with large effect size η^2^ = 0.85).

Regarding GABA levels, the best effects were observed after treatment with PEA-m and NAC compared to H_2_O_2_ at 200 μM (*p* < 0.05). All the new ingredients in IperALA^®^ Forte gave statistically significant results compared to ALA and vitamin D3 (*p* < 0.05).

## 3. Discussion

Neuroprotection plays a crucial role in preventing the transition from acute to chronic pain, as persistent oxidative and inflammatory stress can impair neuronal integrity and synaptic function [51]. Accumulating evidence indicates that prolonged activation of glial cells, excessive production of ROS, and release of pro-inflammatory cytokines contribute to neuronal vulnerability and dysfunction, creating a self-sustaining cycle that exacerbates nociceptive signalling [52]. Within this framework, the maintenance of redox and inflammatory homeostasis emerges as a key factor in preserving neuronal function and preventing maladaptive responses associated with pain persistence. Conventional pharmacological treatments, although effective in reducing pain perception, often fail to address these underlying neurobiological alterations and are limited by long-term adverse effects [53]. Consequently, increasing attention has been directed toward neuroprotective and antioxidant strategies, including nutraceutical formulations and bioactive compounds, aimed at supporting neuronal resilience by modulating OS, inflammation, and neurotransmission balance [53,54].

From this perspective, this study focused on examining a nutraceutical formulation of IperALA^®^, intending to maintain or enhance its effect by replacing ALA and vitamin D3 with new bioactive components (*C. sativum* e.s.; NAC and GSH in IperALA^®^ Forte). Being a formulation for oral use, it was necessary to avoid any cytotoxic effect at the intestinal compartment level before acting on the nervous target. In this study, a 3D in vitro Transwell^®^ model using intestinal Caco-2 cells was employed to analyse and classify substances under controlled conditions, in line with the biopharmaceutical classification system [55,56]. This model offers a useful platform for obtaining preliminary data on intestinal absorption and in vitro biocompatibility. The tested formulation of IperALA^®^, as well as its individual components, did not show cytotoxic effects, as evidenced by sustained cell viability and metabolic activity across all concentrations evaluated. These findings suggest a good level of in vitro biocompatibility within the intestinal environment. Furthermore, all formulations helped preserve epithelial barrier function, as assessed in vitro by TEER, a widely accepted indicator of TJ integrity [57]. The TEER measurements suggest that, under the experimental conditions, epithelial barrier integrity was maintained in vitro, with no evident indications of compromised permeability or structural disruption. The research showed that within the 1 h–6 h range, both IperALA^®^ and IperALA^®^ Forte enhanced the permeability rate of individual agents, peaking at 4 h. Across all analysed parameters, the substitution of ALA with NAC, GSH, and CORILOL 15% (*C. sativum* e.s.) at the intestinal level was associated with improved in vitro biocompatibility and enhanced functional properties of the formulation. These effects may reflect a synergistic interaction among the components, particularly the NAC-GSH combination, which appears to play a key role in the observed improvement. This trend is in line with previous findings [46].

Following the intestinal metabolisation in the 3D Transwell^®^ model, all evaluated substances maintained their bioactive capabilities at the nervous target level. This was validated by traversing a validated 3D model of the BBB with co-culture of HUVECs, astrocytes, and pericytes [58,59]. The role of in vitro BBB models is vital for speeding up the CNS drug development process. Consequently, the area has noted different approaches to create and examine the barrier properties across the BBB using cells sourced from humans. The HUVEC–astrocyte–pericyte co-culture model provided a valuable understanding of how astrocytes contribute to the expression of the BBB feature, facilitating analysis of absorption [58,59,60]. The research showcased how each agent and formulation of the two products studied played a role in preserving cell viability and reducing OS on the BBB. As confirmed at the intestinal level, the barrier function was sustained due to the elevated levels of essential TJ proteins like claudin-5 and marveld. In this stage of analysis, both IperALA^®^ and IperALA^®^ Forte facilitated the identification of a synergistic enhancement effect by the individual components, clearly enhancing the permeability of the substances being examined across the BBB. The immunocytochemistry methodology was followed to obtain data validating the ELISA kit on claudin-5 (described with photos in Appendix A). In comparison to the untreated control condition, the findings demonstrated an increase of 25% and 42% in the signal density of claudin-5 following treatment with IperALA^®^ and IperALA^®^ Forte, respectively. For every parameter analysed, the replacement of ALA with NAC, GSH, and CORILOL 15% (*C. sativum* e.s.) for IperALA^®^ assumes a combined effect. Indeed, these findings, observed at the intestinal level and confirmed by BBB permeability data, suggest a significant interaction among the components of IperALA^®^ and IperALA^®^ Forte in enhancing compound absorption and tissue distribution. Supporting this, literature pharmacokinetic data show that micronised PEA improves intestinal absorption and tissue bioavailability, selectively accumulating in inflamed areas, including the CNS, crossing the BBB with efficiency [24,37]. Micronisation significantly improves its dissolution rate and absorption efficiency by increasing surface area and facilitating uptake via transcellular pathways in the small intestine [37]. At the same time, BCP is rapidly absorbed in the gut and is able to cross the BBB and modulate neuroinflammatory pathways via CB2 receptor activation [39,61,62]. Similarly, in the literature, ALA and vitamin D3 (in IperALA^®^) display good oral bioavailability and BBB penetration, contributing to redox and immune modulation [41,63]. In IperALA^®^ Forte, NAC and GSH enhanced antioxidant defence and demonstrated BBB permeability, as described in the literature [64] especially under OS conditions. The data obtained on in vitro biocompatibility and lack of cytotoxicity at both the intestinal level and across the BBB model, together with the preservation of bioactive properties despite cellular metabolism, supported the transition to CNS-focused investigations. These were conducted using a three-dimensional in vitro model aimed at exploring therapeutic strategies against induced neuroinflammation (via H_2_O_2_ 200 μM) for hypothetical applications in nociception and chronic pain management [65,66].

Regarding the CNS, brain organoids represent a significant advancement in neuroscience, offering a powerful model to study human brain development and the onset of neurological diseases [65,66]. Developed to overcome the limitations of animal models, these three-dimensional structures are derived from iPSCs and form through a combination of specific molecular cues and optimised culture conditions. The resulting organoids are self-organising, multicellular, and functionally resemble human brain tissue, making them highly suitable for investigating homeostasis, tissue regeneration, and the mechanisms underlying various brain disorders [67,68]. In this experimental context, the individual bioactive agents showed measurable effects on specific cellular parameters, such as viability, OS response, and structural integrity, under conditions of H_2_O_2_-induced damage. Notably, both tested formulations demonstrated an enhanced ability to mitigate the detrimental effects associated with OS, suggesting a possible additive or synergistic interaction among their components. This is particularly relevant given that elevated levels of ROS, as modelled by the H_2_O_2_ treatment, have been identified as predisposing factors in the onset of astrogliosis and, commonly, astrocyte-related inflammation [69,70,71].

According to what has been explained, after 24 h of treatment, the pro-inflammatory cytokines TNFα and IL-1β were significantly downregulated in the 3D neuronal organoid model, suggesting a decrease in the neuroinflammatory milieu. Since these cytokines are known to mediate neuronal damage and synaptic dysfunction [72], their reduction indicates a protective adjustment of the redox–inflammatory balance and bolsters the in vitro neuroprotective potential of the tested formulations (IperALA^®^ and IperALA^®^ Forte). The organoid model used, generated via EBs and neural induction in N2/B27, led to the formation of three-dimensional structures containing neurons and glial cells, particularly astrocytes. After approximately 30 days, the system showed a good level of neuroepithelial maturation, but did not include endogenous microglia, as it was derived from mesodermal progenitors absent in the protocol. Exposure to H_2_O_2_ (200 μM) induced an inflammatory response mainly attributable to astrocytes, known to release cytokines (IL-1β, IL-6, TNFα), ROS, and reactive nitrogen species (RNS) in response to OS [70]. Neurons may contribute to a lesser extent by activating secondary signals such as damage-associated molecular patterns [73]. Given the composition and degree of maturation of the model (30 days) [66], it is believed that the inflammatory response observed is predominantly mediated by astrocytes. Although the lack of microglia limits full modelling of the CNS immune axis, it allows clearer analysis of astrocyte-driven responses. Astrocytes play a key role in neuroinflammation and nociception by regulating GABA homeostasis and neuronal excitability [74]. Their expression of CB2R further supports its involvement in anti-inflammatory and neuroprotective mechanisms [75]. The results suggest that the formulation may modulate CB2R and GABA-related molecular pathways involved in neuroinflammation and OS, contributing to neuroprotection and suggesting its role in modulating nociception. In this context, in the molecular profile associated with chronic damage, increased expression levels of CB2R and GABA were observed compared to the H_2_O_2_ 200 μM condition. The substitution of ALA and vitamin D3 with NAC, GSH, or CORILOL 15% (*C. sativum* e.s.) resulted in an enhanced biological response under identical experimental conditions of the formulation, suggesting a synergistic effect (already evident in IperALA^®^). The combined presence of PEA-m and endophyllene 15% (*Piper nigrum* e.s.) was hypothesised to increase levels of CB2R. Indeed, the analgesic role of PEA via modulating the CB2R-PPAR-α-TRPV1 pathway is widely recognised, and at the same time, endophyllene 15% (*Piper nigrum* e.s.) can bind CB2R via BCP, acting as a PEA agonist, positively influencing inflammation and nociception response [76]. At the same time, CB2R is implicated in both GABAergic and glutamatergic pathways, as well as in motivation and cognition [77]. Consequently, it was also essential to concentrate on GABA-A since it has been shown to function as a significant inhibitory neurotransmitter in the CNS of mammalian animals [78]. In vivo studies demonstrated that BCP was capable of modulating mechanisms related not only to pain but also to anxiolytic states involving the GABAergic pathway [39]. At the same time, for the extract of *C. sativum*, a significant effect was found in studies at the nervous level using an in vivo model. Results of real-time reverse polymerase chain reaction (RT-PCR) showed that gene expression of the GABA-A receptor was significantly increased [79]. Our results suggest a combined positive action on both CB2R and GABA-A levels, which were found to be improved and increased compared with IperALA^®^, confirming what was previously described in the scientific literature. Additionally, in IperALA^®^ Forte, the presence of *C. sativum* extract contributed to improving the antioxidant and anti-inflammatory properties of the formulation. Indeed, it has also been demonstrated in the literature that the polyphenols contained in it have a remarkable affinity for modulating the TNFα pathway, contributing to ensuring a favourable environment for neuronal well-being [80] by cooperating with NAC and GSH. The importance of the redox status of thiols in optimising the cell’s ability to protect itself from OS has been well-documented. Regarding GSH availability, the most crucial issue is maintaining appropriate blood cysteine levels, as cysteine is recognised as the limiting substrate for glutathione synthesis [81,82]. It has also been established that NAC is an excellent free radical scavenger, contributing substantially to the maintenance of GSH levels in cells. Numerous studies have demonstrated that NAC can help reduce fatigue or prolong the time of GSH accumulation, as well as prevent the initiation of apoptosis [83,84,85]. NAC can help maintain optimal GSH levels, thereby ensuring the body’s capacity to counteract OS. The findings in the antinociceptive setting are consistent with studies where phytoextracts and nutraceuticals such as PEA have produced optimal in vitro outcomes in terms of nervous well-being in neuronal models with astrocytes [86,87,88].

In conclusion, these results suggest that the formulation may influence molecular pathways associated with neuroinflammation and OS, which are mechanistically linked to nociception and pain modulation, although direct nociceptive data were not assessed in this study. The neuroprotective bioactive effects of IperALA^®^ Forte, as evidenced by increased levels of CB2R and GABA, were maintained after intestinal and BBB metabolism. Indeed, optimal results were obtained in the brain organoid model, thus validating the experimental approach.

Despite employing proven human in vitro models, such as 3D neural organoids, HBMEC, astrocytes for the BBB, and Caco-2 for the intestinal barrier, the results should be cautiously extrapolated to clinical outcomes. Considering their inability to accurately depict the intricacy of the human body, particularly its interactions with the immunological, endocrine, and neurological systems, these models provide a strong and mechanistically applicable foundation for investigating human-specific responses in controlled settings. Consequently, more in vivo and clinical research will be required to validate the bioactive potential of the novel formulation in comparison to the original formulation and to evaluate any physiological modifications that could deviate from those shown in vitro, even if our findings offer insightful mechanistic information.

## 4. Materials and Methods

### 4.1. Agents Preparation

All samples provided by Pharma Suisse Laboratories SpA (Milan, Italy), consisting of two formulations already on the market (IperALA^®^ and IperALA^®^ Forte; Table 1), were compared not only with each other but also with individual agents. For clarity, the test samples are listed in Table 1 along with their corresponding human dosages. All substances tested were dissolved in 1 mL of Dulbecco’s Modified Eagle’s Medium (DMEM, Merck Life Science, Rome, Italy) without phenol red and supplemented with 2 mM L-glutamine (Merck Life Science, Rome, Italy), and 1% penicillin–streptomycin (Merck Life Science, Rome, Italy) for all analyses. Substances were diluted 1:2000 to replicate the human dose in vitro [89]. In a similar manner, H_2_O_2_ (Merck Life Science, Rome, Italy) was also added to the same medium as the other agents under consideration, at a final concentration of 200 µM [58].

### 4.2. Cell Cultures

Caco-2 human intestinal epithelial cells purchased from American Type Culture Collection (ATCC, Manassas, VA, USA) were cultured in Advanced Dulbecco’s Modified Eagle’s Medium/Nutrient F-12 Ham’s (Adv DMEM-F12; GIBCO^®^ ThermoFisher Scientific, Waltham, MA, USA) containing 10% foetal bovine serum (FBS), 2 mM L-glutamine, and 1% penicillin/streptomycin and maintained in a 37 °C incubator at 5% CO_2_ [90]. The research utilised cells with 26 to 32 passages to maintain integrative paracellular permeability and transport properties, similar to those observed in intestinal absorption following oral consumption [90]. To conduct various experiments, cells were plated in various ways. For cell viability, 1 × 10^4^ cells were placed in 96-well plates using a 3-(4,5 Dimethylthiazol-2-yl)-2,5-diphenyltetrazolium bromide (MTT)-based In Vitro Toxicology Assay Kit (Merck Life Science, Rome, Italy). In comparison, 2 × 10^4^ cells were placed in a 24-well plate using a 6.5 mm Transwell^®^ with a 0.4 µm pore polycarbonate membrane insert (Corning Costar, New York, NY, USA) to study absorption and integrity (Trans-Epithelial Electrical Resistance, TEER values) through this intestinal model.

The ATCC (Manassas, VA, USA) provided the human astrocyte cell line CCF-STTG1, which was used in the second phase of the study to examine the biological effects of all materials analysed. The experiment’s cells were isolated from the brain of a 68-year-old patient who had astrocytoma. They were then cultivated in flasks using Roswell Park Memorial Institute medium (RPMI, Merck Life Science, Rome, Italy) supplemented with 10% FBS (Merck Life Science, Milan, Italy), 2 mM Hepes (Merck Life Science, Rome, Italy), 2 mM L-Glutamine (Merck Life Science, Rome, Italy), and 1% P/S (Merck Life Science, Rome, Italy). When the cells reached 75% to 85% confluence, they were employed for studies at passage 2 [91]. To replicate the blood–brain barrier (BBB) in vitro model, astrocytes were co-cultured with human umbilical vein endothelial cells (HUVECs) and human cerebral vascular pericytes (HBVPs) according to procedures documented in the literature [92].

ScienCell Research Laboratories’ HBVP cells were cultured in a pericyte medium supplemented with a growth supplement and 2% FBS (ScienCellTM Research Laboratories, Carlsbad, CA, USA).

HUVECs were bought from ATCC (Manassas, VA, USA). Cells were grown in Endothelial Cell Growth Medium (EGM Media, Lonza, Basel, Switzerland) supplemented with 2% FBS, hydrocortisone (0.04%), human fibroblast growth factor beta (0.4%), vascular endothelial growth factor (0.1%), recombinant human insulin-like growth factor I analogue (0.1%), ascorbic acid (0.1%), human epidermal growth factor (0.1%), Gentamicin sulphate–Amphotericin (0.1%), and heparin (0.1%) (all reagents from Lonza, Walkersville, MD, USA). Between passages three and six, cells were maintained at 37 °C in an atmosphere with 5% CO_2_ and 95% humidified air. To form the BBB, 3 × 10^5^ cells/well were seeded on a Transwell^®^ insert in co-culture with pericytes and astrocytes as described in Section 4.6 following a protocol found in the literature [93].

The iPSCs were bought from the ATCC (Manassas, VA, USA) and maintained in appropriate CO_2_ and culture medium conditions for the preparation of the brain organoid model described in Section 4.11.

### 4.3. Experimental Protocol

**Figure 7:**
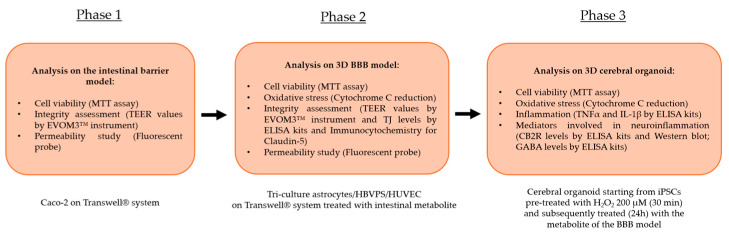
An illustration of the flow of the several stages that make up the experimental procedure and the studies performed.

The experimental study consisted of different analyses and methods distributed in 3 distinct phases characterised by specific cellular compartments subjected to stimulation with the test samples (Figure 7).

The first phase of the study focused on analysing the effects of the individual agents, the commercial product, and the new formulation at the level of an in vitro intestinal barrier model on the Food and Drug Administration (FDA) and European Medicines Agency (EMA)-approved Transwell^®^ system. Specifically, after treatment for 1 h to 6 h, analyses were performed on the maintenance of cell viability by the MTT assay to avert any cytotoxic effects at the intestinal level. Accurate measurement of TEER by an EVOM3™ instrument was essential at this level; this was to study the maintenance of intestinal barrier integrity status. Finally, during the 1 h–6 h treatment period, the permeation status along the intestinal barrier of the various samples under examination was studied by a fluorescent probe. Then the intestinal metabolites from each test sample present at the level of the lower or basolateral compartment of the Transwell^®^ system were collected. This allowed the peripheral blood to be repurposed in vitro and contained specifically the intestinal metabolites and the substances that correctly crossed the intact epithelium. The supernatant was used as a stimulus for its subsequent cellular target following the correct physiological process after intestinal assimilation. The intestinal metabolites from each sample, contained at the level of the lower or basolateral compartment of the Transwell^®^ system, were collected and used as a stimulus for the next phase with the BBB model (Phase 2).

In the second phase of the experimental protocol, analyses were performed at the level of a BBB tri-culture model composed of a co-culture of astrocytes with HBVPS and HUVEC cell lines. In this phase following treatment for 24 h with the intestinal metabolites associated with individual agents, IperALA^®^ and IperALA^®^ Forte were used in the following analyses: the possible appearance of cytotoxic effects through an MTT viability test and OS analysis by cytochrome C; the state of integrity of the BBB using specific ELISA kits to detect the main TJ or Claudin-5 and MARVELD; analysis of the rate of absorption (at 15′–30′–1 h–3 h–12 h–24 h) using a fluorescent probe as performed at the intestinal level. Furthermore, for Claudin-5, additional analyses of its presence were performed through an immunocytochemistry protocol with collection of representative images following treatment with IperALA^®^ and IperALA^®^ Forte (see Appendix A).

At this point, each sample’s supernatant from the basolateral compartment was collected. This was performed to gather the cellular metabolites and chemicals that had successfully crossed the BBB. This was employed as a stimulus in the subsequent phase for cerebral organoids.

The third and final phase of the research focused on the analysis of the effects of samples on the CNS. The cellular metabolites from the BBB model of each sample were used as a treatment for 24 h on an organoid model of the brain with 200 μM H_2_O_2_-induced OS conditions as described in the literature to recreate the conditions of pain and inflammation [58]. The following analyses were carried out at this stage: cell viability by the MTT test; OS by cytochrome C; protein markers associated with inflammation (TNFα and IL-1β) by specific ELISA kits and markers related to the triggering of neuroinflammation and the context of nociception and pain such as CB2R and GABA levels by ELISA kits. At the same time, CB2R presence was also characterised using Western Blots.

### 4.4. In Vitro Intestinal Barrier Model

Employing the Transwell^®^ system, an in vitro intestinal barrier model was established by a standard procedure documented in the scientific literature to determine whether the substances utilised can pass through the intestinal barrier [94] in accordance with protocols approved by the FDA and the EMA [95,96]. These procedures are implemented to predict the absorption, metabolism, and bioavailability of various substances following oral administration in humans. In short, for 21 days before stimulation, Caco-2 cells plated on a Transwell^®^ insert were maintained in full medium, which was replaced every other day at both the basolateral and apical sides [97]. Throughout the whole development period, the TEER values were measured using EVOM3™ and STX2 chopstick electrodes (World Precision Instruments, Sarasota, FL, USA) to examine the development of mature intestinal epithelium and an appropriate paracellular mechanism. On the 21st day, when TEER readings were more than 400 Ω∙cm^2^, absorption analysis proceeded [94]. The culture medium’s apical-side pH of 6.5, which is similar to the pH in the small intestine lumen, was confirmed before stimulation. On the basolateral side, however, pH 7.4 denoted blood [98]. From 1 to 6 h before the subsequent analyses, the cells were stimulated with all compounds. A fluorescent tracer at a concentration of 0.04% (Santa-Cruz, CA, USA) was used to detect the permeability rate at each time point [90]. Caco-2 cells were incubated for 40 min at the previously mentioned concentration to quantify the amount of fluorescein transported at 37 °C. A fluorescence spectrophotometer (Infinite 200 Pro MPlex, Tecan, Männedorf, Switzerland) was used to detect fluorescence at excitation and emission wavelengths of 490 and 514 nm, respectively. The following formula was used to obtain the permeation rate [99]:

J = Jmax [C]/(Kt + [C])(1)

where

- Jmax: the maximum permeation rate;
- [C]: the initial concentration of fluorescein;
- Kt: the Michaelis–Menten constant.

Results are expressed as mean ± SD (%). Negative controls without cells were tested to exclude the influence of the Transwell^®^ membrane.

### 4.5. Cell Viability (MTT Test)

Applying the MTT In vitro Toxicology Assay Kit (Merck Life Science, Rome, Italy), cell viability was verified on every experimental protocol phase following each stimulation in accordance with a standard procedure reported in the literature [92]. All solubilised samples, both treated and untreated, had their absorbance measured at 570 nm with correction at 690 nm using a spectrometer (Infinite 200 Pro MPlex, Tecan, Männedorf, Switzerland). The control sample and the data were contrasted. The findings are shown as the average ± standard deviation (%) of viable cells relative to the control (untreated samples) of five independent experiments performed in triplicate.

### 4.6. Blood–Brain Barrier (BBB) In Vitro Model

The astrocyte cell line was co-cultured with HBVPS and HUVEC lines in accordance with the procedure outlined in the literature [93]. A tri-culture Transwell^®^ model of the BBB was created using 12-well Transwell^®^ inserts. Type I collagen (150 µg/mL; Merck, Darmstadt, Germany) and poly-L-lysine (15 µL; ScienCell, Carlsbad, CA, USA) were applied to inserts, incubated for two hours at 37 °C, cleaned with DPBS (Merck Life Science, Rome, Italy), and allowed to air dry in a sterile environment. For two to three hours, CCF-STTG1 cells (3.13 × 10^5^ cells/well) were incubated at 37 °C in 5% CO_2_ after being sown on the basolateral side. After inverting the inserts, any excess media were taken out and reinserted into both sections. After that, any excess media were taken out and the inserts were inverted. The apical side of each compartment received 800 µL of fresh culture media, whereas the basolateral side received 1600 µL. To protect the cell layer, the astrocyte media was carefully removed from the plates after they had been incubated for 48 h. Following this, inserts were reversed for further processes. At a density of 6.25 × 104 cells/well, or around a 5:1 astrocyte-to-pericyte ratio, HBVPs were directly seeded into the astrocyte layer. Throughout another two to three hours of incubation, the implants were repositioned, extra medium was sucked, and a 1:1 combination of astrocyte and pericyte media was introduced to each compartment. The co-culture was maintained under these conditions until approximately 90% confluency was achieved, typically by day 4.

After removing the apical media, HUVECs were seeded at a density of 3 × 10^5^ cells/well onto the collagen-coated apical surface of the inserts. Before the medium was changed, the cells were left to adhere for at least five hours at 37 °C. An EVOM3^TM^ voltohmmeter was used to quantify TEER following a 24 h HUVEC seeding period. The measured resistance (in ohms) was multiplied by the Transwell^®^ insert’s surface area to determine the TEER values, which were then expressed in Ω × cm^2^. This configuration allows for the assessment of barrier integrity and cellular interactions in regulated in vitro settings by simulating important structural and functional aspects of the human BBB. Only for additional analyses with immunocytochemistry (Section 4.9) was the 3D model of the BBB set up on slide chambers.

Treatment and permeability investigations were performed on Transwells^®^ after 7 days of culture [99]. The test compounds were applied to cells for 15 min to 24 h before BBB permeability was measured with a fluorescent marker. Fluorescein (0.04%; Merck Life Science, Rome, Italy) was used to quantify basolateral compartment fluorescence using a spectrophotometer (Infinite 200 Pro MPlex, Tecan, Männedorf, Switzerland) with excitation/emission settings of 490/514 nm. Values were represented as a percentage of the starting chemical that crossed the cell layer, determined using the formula outlined in reference [46]:

J = Jmax [C]/(Kt + [C])(2)

where

- Jmax: the maximum permeation rate;
- [C]: the initial concentration of fluorescein;
- Kt: the Michaelis–Menten constant.

Results are expressed as mean ± SD (%), and negative controls without cells were tested to exclude influence by the Transwell^®^ membrane.

### 4.7. Reactive Oxygen Species (ROS) Production

The rate of superoxide anion release in the BBB and cerebral organoid in vitro models was measured using a conventional method based on cytochrome C reduction [100]. For 30 min, both treated and untreated cells were incubated with 100 µL of cytochrome C and 100 µL of superoxide dismutase (both supplied by Merck Life Science, Rome, Italy). The absorbance in culture supernatants was measured at 550 nm using a spectrometer (Infinite 200 Pro MPlex, Tecan, Männedorf, Switzerland). O_2_ is defined as the mean ± SD (%) of nanomoles of reduced cytochrome C per microgram of protein in comparison to the control (0 line) of five separate experiments carried out in triplicate.

### 4.8. Claudin 5 Assay Kit

The quantity of claudin 5 in BBB cell lysates was measured using the Claudin 5 ELISA Kit (MyBiosource, San Diego, CA, USA) according to the manufacturer’s instructions [101]. The absorbance was measured at 450 nm using a spectrometer (Infinite 200 Pro MPlex, Tecan, Männedorf, Switzerland). The concentration was expressed in ng/mL by comparing the results to the standard curve (from 0 to 2500 pg/mL) and was shown as mean values ± SD (%) relative to the control (untreated cells) of five independent experiments performed in triplicate.

### 4.9. Tricellulin (MARVELD Protein) Assay Kit

The tricellulin/MARVELD in BBB cell lysates was measured using a Tricellulin ELISA Kit (MyBiosource, San Diego, CA, USA) according to the producer’s protocol [58]. Absorbance was measured at 450 nm using a spectrometer (Infinite 200 Pro MPlex, Tecan, Männedorf, Switzerland). The concentration was calculated by comparing the data to the standard curve (0.625 to 20 ng/mL). It is expressed as mean values ± SD (%) relative to the control (untreated cells) of five independent experiments performed in triplicate.

### 4.10. Claudin 5 Immunocytochemistry in Cellular Preparation

The chamber slide used to prepare the BBB tri-culture model was washed three times with cold PBS 1X supplemented with 2 mM sodium orthovanadate. It was then fixed for 20 min at room temperature (RT) using a cold fixative solution (3.7% formaldehyde, 3% sucrose in PBS 1X). Following this, the cells were permeabilised with cold PBS 1X using cold 0.5% Triton X-100 on ice at 4 °C for 20 min. This was followed by two PBS 1X washes. All chemicals described above were purchased from Sigma-Aldrich (Milan, Italy). After blocking endogenous peroxidase activity for 8 min with 3% H_2_O_2_ in PBS 1X, the chamber slides were kept in a blocking solution made of PBS 1× and 3% albumin from bovine serum (BSA, Sigma-Aldrich, Milan, Italy) for 1 h at room temperature. A particular primary antibody, 1:150 Claudin-5 (Santa Cruz Biotechnology, Santa Cruz, CA, USA), was then added to the slides and incubated for the whole night at 4 °C. In a humidified chamber, the antibody was diluted in PBS 1×. It was then incubated for 20 min with a diluted biotinylated secondary antibody solution (Dako Italia, Milan, Italy) and with VECTASTAIN^®^ ABC Reagent (ThermoFisher Scientific, Waltham, MA, USA) for another 20 min. Following a final wash, the sections were counter-stained with Mayer’s hematoxylin, incubated with peroxidase substrate solution until the appropriate stain intensity developed (Peroxidase/DAB, Dako Italia, Milan, Italy), rinsed in tap water, and mounted using Bio Mount (Bio-Optika, Milan, Italy). The claudin-5 expression density in terms of number of positive cells was computed using the method outlined in the literature [102,103]: briefly, 12 different areas (1 mm^2^) randomly selected from each section were taken, and the number of signals was determined using ImagePro 3 software (NIH, Bethesda, MD, USA) [103]. The results were expressed as a means ± SD (%) and reported in Appendix A.

### 4.11. In Vitro Model of Brain Organoid

According to a protocol reviewed in the literature [66], the process began with the selection of iPSCs (ATCC, Manassas, VA, USA). These cells were cultured in StemFlex medium (ThermoFisher Scientific, Waltham, MA, USA) on Matrigel-coated plates (Corning, USA) under standard conditions in a CO_2_ incubator. Pluripotency was maintained with basic fibroblast growth factor (bFGF, PeproTech, ThermoFisher Scientific, Waltham, MA, USA) and ROCK inhibitor Y27632 (Merck, Germany), and regular testing was performed to confirm the absence of mycoplasma contamination. The differentiation process began with the formation of embryoid bodies (EBs) by seeding 2 × 10^3^–18 × 10^3^ cells per well in ultra-low-attachment 96-well U-bottom plates (Corning, USA) using Dulbecco’s Modified Eagle’s Medium/Nutrient F-12 Ham’s (DMEM-F12, GIBCO^®^ ThermoFisher Scientific, Waltham, MA, USA) supplemented with Knockout Serum Replacement (KSR), GlutaMAX, MEM non-essential amino acids (NEAA), and heparin (Sigma-Aldrich, USA). EBs were cultured in Neural Induction Medium, containing N2 supplement (ThermoFisher Scientific, Waltham, MA, USA), to promote neuroectodermal differentiation. By day 5–6, neuroepithelial structures became visible, and EBs were transferred to low-attachment 24-well plates in the same medium for further maturation. By day 11–12, neural aggregates were embedded in growth factor-reduced Matrigel (Corning, USA) and transferred to Petri dishes. This embedding step is crucial for maintaining the integrity of the neuroepithelial buds and preventing premature differentiation. After 1–2 additional days, the aggregates exhibited radially organised neuroepithelial structures, indicative of successful neural induction. At day 14–18, the organoids were transferred to an orbital shaker (MPS-200, angzhou Haipei Instrument Co., Ltd., Hangzhou City, China) for continued differentiation in Improved Differentiation Medium (IDM-A) without vitamin A, containing Neurobasal medium, N2 supplement, B27 supplement without vitamin A, insulin (Merck, Germany), GlutaMAX, and penicillin–streptomycin (ThermoFisher Scientific, Waltham, MA, USA). The agitation provided by the orbital shaker (set to 57 rpm) ensured proper diffusion of nutrients and oxygen, preventing central necrosis. At day 30, neuronal maturation was enhanced by transitioning to IDM + A medium, which includes B27 supplement with vitamin A and dissolved Matrigel to support extracellular matrix interactions. The presence of ascorbic acid (Merck, Germany) and sodium bicarbonate supported oxidative metabolism and cellular health.

### 4.12. TNFα Production ELISA Kit

TNFα measurement in cerebral organoid model supernatants was performed using the TNFα ELISA kit (Merck Life Science, Rome, Italy) according to the manufacturer’s guidelines [104]. The samples’ absorbance was measured at 450 nm with a plate reader (Infinite 200 ProMPlex, Tecan, Männedorf, Switzerland). The outcomes were obtained utilising a calibration curve (range: 24.58 pg/mL–6000 pg/mL) and are presented as mean values ± SD (%) relative to the control (0 line) from five independent experiments conducted in triplicate.

### 4.13. IL-1β Production ELISA Kit

IL-1β levels in cerebral organoid lysates were measured according to the manufacturer’s guidelines with an IL-1β ELISA Kit (R&D Systems, Minneapolis, MN, USA). The absorbance was assessed using a microplate reader at 450 nm, with a correction at 570 nm. The quantification of IL-1β involved comparing the sample readings with the standard curve (3.9–250 pg/mL) created [105]. Results are presented as mean values ± SD (%) relative to the control (0 line) from five independent experiments conducted in triplicate.

### 4.14. CB2R ELISA Kit

The CNR2 ELISA Kit (FineTest, Wuhan, China) was utilised in cerebral organoid supernatants, adhering to the manufacturer’s guidelines to confirm the functionality of the CB2 receptor [106]. The plate was analysed at 450 nm utilising a plate reader (Infinite 200 Pro MPlex, Tecan, Männedorf, Switzerland). The data were collected and analysed against the standard curve (spanning from 62.5 to 4000 pg/mL). The outcomes were reported as mean ± SD (%) compared to the control (0 line) from five separate experiments conducted in triplicate.

### 4.15. GABA ELISA Assay

The GABA ELISA Kit (FineTest, Wuhan, China) was used on cerebral organoid lysates following the manufacturer’s instructions [107]. The absorbance was measured at 450 nm through a plate reader (Infinite 200 ProMPlex, Tecan, Männedorf, Switzerland). The data were obtained and compared to the standard curve (6 to 400 pg/mL). The results were expressed as mean ± SD (%) versus control (0 lines) of five independent experiments in triplicate.

### 4.16. Western Blot

The cerebral organoid was lysed on ice using Complete Tablet Buffer (Roche, Basel, Switzerland) supplemented with 1 mM PMSF, 2 mM sodium orthovanadate (Na_3_VO_4_), a 1:50 dilution of phosphatase inhibitor cocktail, and a 1:200 dilution of protease inhibitor cocktail, following a standard protocol [97]. Protein extracts (35 µg) were subjected to separation via 10% Sodium Dodecyl Sulphate–Polyacrylamide Gel Electrophoresis (SDS-PAGE), followed by transfer onto polyvinylidene fluoride (PVDF) membranes (GE Healthcare Europe GmbH, Milan, Italy). Membranes were incubated overnight at 4 °C with primary antibodies diluted at 1:500 (Santa Cruz Biotechnology, Santa Cruz, CA, USA) that specifically target CB2R. Protein loading was assessed using an anti-β-actin antibody diluted to 1:4000 (Merck Life Science, Rome, Italy). Data were presented as mean ± SD (%) compared to the untreated control.

### 4.17. Statistical Analysis

All results were expressed as mean ± SD of at least five independent biological replicates, each performed in technical triplicate. For each experimental protocol, data was normalised to the untreated control, consisting of cells exposed only to the culture medium (i.e., without formulations or individual components), which was used as a reference.

Data normalisation was performed by dividing the mean optical density (OD) of each treated sample (calculated from technical triplicates) by the mean OD of the untreated control sample, which consisted of cells incubated with culture medium alone (i.e., without any formulations or components) and served as the baseline reference.

The resulting ratios were then converted into percentage variation relative to the control by subtracting 1 (the normalised control value) and multiplying by 100, according to the formula

$$\%\;\mathrm{variation}\;=(\frac{\;\mathrm{mean}\;\mathrm{OD}\;\mathrm{treated}}{\;\mathrm{mean}\;\mathrm{OD}\;\mathrm{control}}-1)\times 100$$

This normalisation approach sets the untreated control at 0%, enabling straightforward interpretation of treatment-induced increases or decreases in viability/metabolic activity across all datasets.

Statistical comparisons between groups were performed using one-way ANOVA followed by Bonferroni’s post hoc test, or the Mann–Whitney U test when appropriate. Data collected at different time points (such as TEER values, viability) were analysed separately by one-way ANOVA followed by Tukey’s post hoc test. This approach is used for measurements taken at different times from independent biological replicates. Consequently, each time point was treated as an independent experiment to evaluate treatment-dependent differences under identical temporal conditions. All analyses were conducted using GraphPad Prism version 10.2.3 (GraphPad Software, La Jolla, CA, USA). A *p*-value < 0.05 was considered statistically significant. The effect size between the two formulations under consideration was quantified using η^2^ (eta squared). In GraphPad Prism, η^2^ corresponds to the R^2^ value reported in the ANOVA table and is calculated as the ratio of the between-group sum of squares to the total sum of squares (η^2^ = $\frac{{\mathrm{SS}}_{\mathrm{between}}}{{\mathrm{SS}}_{\mathrm{total}}}$). In parallel and with similar results, η^2^ was calculated using the standard formula $\eta^{2}=\frac{t^{2}}{t^{2}+df}$, which provides an equivalent measure of explained variance (pairwise *t*-test) [108].

## 5. Conclusions

The present findings indicate that the IperALA^®^ Forte formulation may exert protective effects on neuronal cells by supporting viability, maintaining structural integrity, and attenuating oxidative and inflammatory stress in 3D in vitro models. Compared with the ALA and vitamin D3-based formulation, the combination of NAC, GSH, and *C. sativum* extract appeared to promote a more favourable redox–inflammatory balance, accompanied by modulation of CB2R and GABA expression. These observations point toward a potential neuroprotective, antioxidant and anti-neuroinflammatory profile, although their biological and translational relevance should be interpreted with caution. Further investigations in advanced in vivo and clinical models are required to confirm these preliminary results and to clarify whether such neuroprotective mechanisms could also contribute to the modulation of nociceptive and pain-related pathways.

## Figures and Tables

**Table 1:** Composition of the formulations with declared human dosages (Dosage) and corresponding in vitro concentrations (In vitro), reflecting a 2000-fold dilution of each component dosage.

IperALA^®^			IperALA^®^ FORTE		
**Sample**	**Dosage**	**In Vitro**	**Sample**	**Dosage**	**In Vitro**
Palmitoylethanolamide micronised (PEA-m)	600 mg	300 μg	Palmitoylethanolamide micronised (PEA-m)	600 mg	300 μg
Endophyllene (*Piper nigrum* e.s.) of which: BCP	50 mg 15 mg	25 μg 7.5 μg	Endophyllene (*Piper nigrum* e.s.) of which: BCP	166.7 mg 50 mg	83.35 μg 25 μg
Vitamin D3 500.000 UI/g	1000 UI	0.5 UI	CORILOL^®^ 15% (*C. sativum* e.s.)	133.4 mg	66.7 μg
ALA 30–60 Mesh	800 mg	400 μg	NAC USP	600 mg	300 μg
			GSH	200 mg	100 μg
Total	1300 mg		Total	3800 mg	

## Data Availability

The data presented in this study are available on request from the corresponding author (the Laboratory of Physiology carefully stores raw data to ensure permanent retention under a secure system).

## Abbreviations

The following abbreviations are used in this manuscript:

Adv DMEM-F12	Advanced Dulbecco’s Modified Eagle’s Medium/Nutrient F-12 Ham’s
Adv-DMEM	Advanced Dulbecco’s Modified Eagle’s Medium
ALA	Alpha-Lipoic Acid
ATCC	American Type Culture Collection
BBB	Blood–Brain Barrier
BCP	β-Caryophyllene
bFGF	Basic fibroblast growth factor
*C. sativum*	*Coriandrum sativum*
CB1R	Cannabinoid Receptor 1
CB2R	Cannabinoid Receptor 2
CNS	Central Nervous System
DMEM	Dulbecco’s Modified Eagle’s Medium
DMEM-F12	Dulbecco’s Modified Eagle’s Medium/Nutrient F-12 Ham’s
EB	Embryoid Bodies
EGM	Endothelial Cell Growth Medium
EMA	European Medicines Agency
FBS	Foetal Bovine Serum
FDA	Food and Drug Administration
GABA	Gamma-Aminobutyric Acid
GSH	Glutathione
HUVEC	Human Umbilical Vein Endothelial Cells
IDM	Improved Differentiation Medium
IL-1β	Interleukin 1β
iPSC	Induced Pluripotent Stem Cells
MTT	3-(4,5-Dimethylthiazol-2-yl)-2,5-Diphenyltetrazolium Bromide
NAC	N-Acetylcysteine
NEAA	Non-Essential Amino Acids
OS	Oxidative Stress
PEA	Palmitoylethanolamide
PEA-m	Palmitoylethanolamide micronised
PNS	Peripheral Nervous System
PPAR-α	Peroxisome proliferator-activated receptor α
RNS	Reactive Nitrogen Species
ROS	Reactive Oxygen Species
RPMI	Roswell Park Memorial Institute medium
TEER	Trans-Epithelial Electrical Resistance
TJ	Tight junction
TNFα	Tumour Necrosis Factor α
